# Indigenous coated needle for nerve block

**DOI:** 10.4103/0019-5049.79874

**Published:** 2011

**Authors:** Dilip Kothari, Jitendra Agrawal, Amrita Mehrotra

**Affiliations:** Department of Anaesthesiology, GR Medical College, Gwalior, Madhya Pradesh, India

Sir,

Success rate of any nerve block highly depends upon precise localization of the nerve, which also reduces the amount of local anaesthetic agent required. Elicitation of paraesthesia with or without the use of nerve locator,[1] and recently the aid of ultrasound[2] are in use for exact localization of nerve.

Injury to nerve, multiple pricks, large amount of local anaesthetic, and high incidence of incomplete block are the main disadvantages of elicitation of paraesthesia technique,[3] whereas the high cost and expertise to use the ultrasound machine are the main deterrent factors for its routine use.

Although conventional needle has been used in the past,[4] but uncoated needle for nerve block are not reliable and safe because applied current is dispersed all over giving inaccurate localization. In contrast coated needle requires a low threshold current because current density focuses mainly at the tip.[5] Modified tuohy needle[6] too has been used for continuous plexus block, but big size needle and difficult sterilization by ethylene oxide are the main disadvantages to its use.

Due to high cost and unavailability of teflon or polymer-coated needles in our hospital, we were in search of a cost-effective alternative. We tried intravenous cannula but application of the current probe was a problem because of full-length teflon coating.

We made an indigenous version with two locally available intravenous cannula (KETHIN^TM^) size 18 and 20 SWG [Figure 1]. Under all sterile condition stylet of size 18 cannula was inserted into size 20 outer Teflon sheath with a gentle force. Due to this force teflon sheath gets detached from its junction at wings (point A), if not then we made a cut at this junction with a sterile blade so the sheath advances tightly on bigger size stylet leaving approximately 2-3 cm of bare area over metal stylet (between point A and B, respectively) where we attached crocodile clip of nerve locator. Then with a sterile surgical blade we removed excess teflon sheath at bevel level to leave the tip bare [Figure 2].

**Figure 1:**
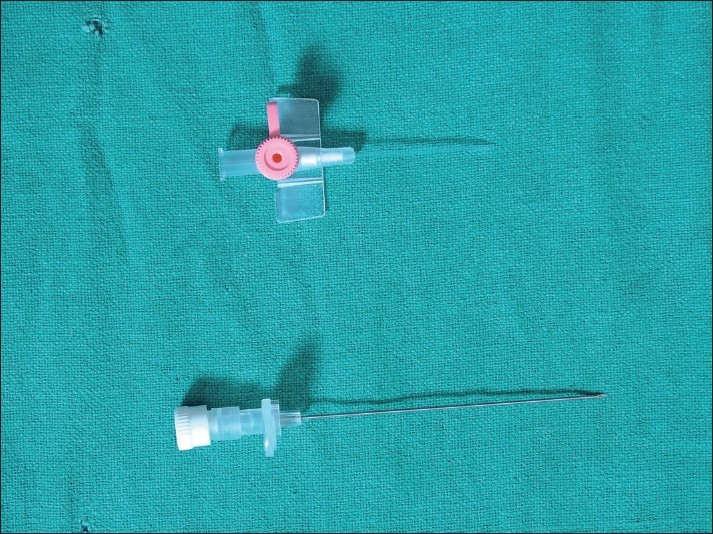
SWG 20 outer teflon sheath and SWG 18 metal stylet of conventional intravenous cannula

**Figure 2:**
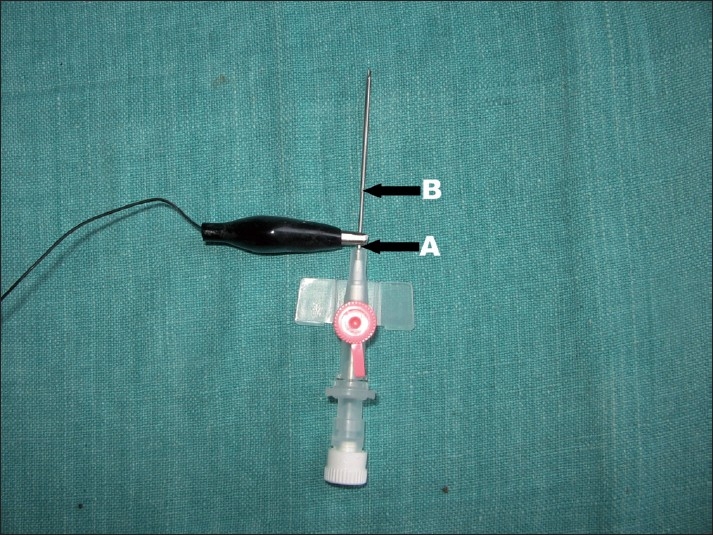
Indigenously prepared coated needle for nerve block along with crocodile nerve locator clamp at bare area between two arrows

This version cost us approximately INR 30-35 (0.5-0.7 Euro) only, whereas the coated needles in market costs approximately INR 500-800 (8-14 Euro). We have been using it regularly for last 6 months with accurate and safe localization of supraclavicular brachial plexus giving good success rate.

## References

[1] Sia S, Bartoli M, Lepri A, Marchini O, Ponsecchi P (2000). Multiple injection axillary plexus block: A comparison of two methods of nerve localization nerve stimulation versus paraesthesia. Anaesth Analg.

[2] Marhofer P, Greher M, Kapral S (2005). Ultrasound guidance in regional anaesthesia. Br J Anaesth.

[3] Kaufman BR, Nystrom E, Nath S, Foucher G, Nystrom A (2000). Debilitating chronic pain syndromes after presumed intraneural injections. Pain.

[4] Furukawa M, Nakagawa K, Hamada T (1994). Brachial plexus block using a nerve stimulator and a conventionalneedle. Osaka City Med J.

[5] Balavenkatsubramanian J (2008). Continuous peripheral nerve block. The future of regional anaesthesia. Ind J Anaesth.

[6] Kulkurni AH (2009). A Modification of Tuohy Needle for continuous plexus blockade. Ind J Anaesth.

